# Surgical technique of a transcutaneous osseointegration prosthesis system (TOPS) for transtibial amputees

**DOI:** 10.1007/s00064-025-00888-8

**Published:** 2025-02-19

**Authors:** Jan Paul Frölke, Ruud Leijendekkers, Robin Atallah

**Affiliations:** 1 Surgery 618, Radboud University Medical Center, Geert Grooteplein Zuid 10, 6525 GA Nijmegen, The Netherlands; 2 AOFE Clinics, Oosterbeek, The Netherlands; 3 Radboud University Medical Center, Nijmegen, The Netherlands; 4 Maartenskliniek, Nijmegen, The Netherlands

**Keywords:** Osseointegration implant, Bone-anchored prosthesis, Transtibial amputation, Below-knee amputation, Rehabilitation, Implantat mit Osseointegration, Knochenverankerte Prothese, Unterschenkelamputation, Amputation unterhalb des Knies, Rehabilitation

## Abstract

Transcutaneous osseointegration prosthetic systems (TOPS) are intended to provide stable skeletal attachment for artificial limbs after extremity amputation and is an alternative for socket attachment. TOPS for individuals with limb amputation using osseointegration implants (OI) has proven to consistently and significantly improve quality of life and mobility for the vast majority of amputees, previously using a socket prosthesis also experiencing socket-related problems. As with any implant, complications such as infection, aseptic loosening, or implant fracture can occur, which may necessitate hardware removal. Approximately half of patients who undergo a below-knee amputation are able to utilize an artificial leg acceptably well with a socket-suspended prosthesis. However, the other half of patients experience limitations resulting in reduced prosthesis use, mobility, and quality of life. Limb-to-prosthesis energy transfer is poor because of the so-called “pseudojoint” (i.e., the soft tissue interface), and gross mechanical malalignment is common. Furthermore, transtibial amputees may experience irritation from pistoning and suction at the residual limb–socket interface. These issues result in skin problems and difficulties with socket fit because of fluctuation in the size of the residual limb size, resulting in a decrease in overall satisfaction and confidence in mobility. A bone-anchored implant creates a direct skeletal connection between the residual limb and artificial leg, in which energy transfer is optimal and mechanical alignment is significantly improved.

## Abstract (de)

Transkutane osseointegrierte Prothesensysteme (TOPS) sollen nach einer Extremitätenamputation eine stabile skelettale Befestigung für künstliche Gliedmaßen bieten und sind eine Alternative zur Schaftbefestigung. Für Personen mit Gliedmaßenamputation, die Osseointegrationsimplantate (OI) verwenden, haben sich TOPS konsistent und signifikant als Verbesserung von Lebensqualität und Mobilität erwiesen: für die überwiegende Mehrheit der Amputierten, die zuvor eine Schaftprothese verwendet und Probleme mit dem Schaft hatten. Wie bei jedem Implantat können Komplikationen, wie Infektionen, aseptische Lockerungen oder Implantatbrüche, auftreten, die eine Entfernung des Implantats erforderlich machen. Etwa die Hälfte der unterschenkelamputierten Patienten ist in der Lage, ein künstliches Bein mit einer schaftgetragenen Prothese zufriedenstellend zu nutzen, doch bei anderen Patienten kommt es zu Einschränkungen, die zu einer verminderten Nutzung der Prothese sowie zur Einschränkung von Mobilität und Lebensqualität führen. Die Energieübertragung von der Gliedmaße zur Prothese ist aufgrund des „Pseudogelenks“ (d. h. der Weichteilschnittstelle) schlecht, grobe mechanische Fehlstellungen sind häufig. Darüber hinaus kann es zu Irritationen durch Hin- und Herbewegen und Sog an der Stumpf-Prothesenschaft-Schnittstelle kommen. Diese Probleme führen zu Hautproblemen und Schwierigkeiten mit dem Sitz des Schafts aufgrund von Schwankungen in der Stumpfgröße und konsekutiv zu einer verringerten Gesamtzufriedenheit und einer Abnahme des Vertrauens in die Mobilität. Ein knochenverankertes Implantat schafft eine direkte skelettale Verbindung zwischen Stumpf und Prothese, bei der die Energieübertragung optimal ist und die mechanische Ausrichtung deutlich verbessert wird.

## Introductory remarks

**Fig. 1:**
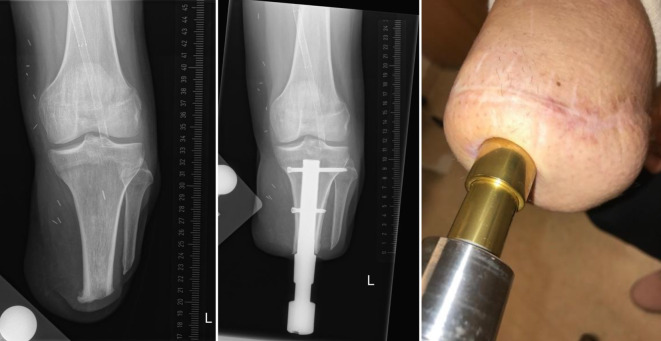
Before (left) and after (middle) implantation of bone-anchored prosthesis with stoma (right)

Transcutaneous osseointegration prosthetic systems (TOPS) are intended to provide a stable skeletal attachment for artificial limbs after extremity amputation and is an alternative for socket attachment. TOPS for individuals with limb amputation using osseointegration implants (OI; Fig. 1) has proven to consistently and significantly improve the quality of life and mobility for the vast majority of amputees, previously using a socket prosthesis also experiencing socket-related problems. As with any implant, complications such as infection, periprosthetic fracture, aseptic loosening, or implant fracture can occur, which may necessitate hardware removal.

Approximately half of patients who undergo a below-knee amputation are able to utilize an artificial leg acceptably well with a socket-suspended prosthesis [4, 10]. However, the other half of patients experience limitations resulting in reduced prosthesis use, mobility, and quality of life. Limb-to-prosthesis energy transfer is poor because of the so-called “pseudojoint” (i.e., the soft tissue interface), and gross mechanical malalignment is common. Furthermore, transtibial amputees may experience irritation from pistoning and suction at the residual limb–socket interface. These issues result in skin problems and difficulties with socket fit because of fluctuation in the size of the residual limb size, resulting in a decrease in overall satisfaction and confidence in mobility. A bone-anchored implant creates a direct skeletal connection between the residual limb and artificial leg, in which energy transfer is optimal and mechanical alignment is radically improved. Since 2014 we have successfully treated about 150 individuals with bone-anchored prosthesis after below-knee amputation [1, 2, 6, 10].

## Surgical principle and objective

The cylindrical BADAL X (OTN Implants Arnhem The Netherlands) tibia implant with a distal drop shape is transcutaneously implanted using a retrograde press-fit approach. Press fit is created with an implant diameter that is 0.6–1.0 mm larger than the surgically prepared intramedullary canal of the humerus and also depending on the individual bone quality. The distal part of the BADAL X tibia implant is provided with a taper connection to the BADAL X dual-cone adapter that serves as an abutment for the fixation of the leg prosthesis. The leg prosthesis can be connected to the BADAL X dual-cone adapter with a compatible quick attach and release ‘Luci’ connector.

## Advantages

- Better prosthetic control and stability/safety
- Prosthetic “feeling”
- Perfect suspension in swing phase
- Better sitting comfort and hip range-of-motion
- No loosening of prosthesis during daily activities
- Donning and doffing is quick and easy
- Stump muscles remain fit
- No sweating in summer

## Disadvantages

- Unnatural skin opening (stoma)
- Life-long stoma care required
- Soft tissue infection in the skin penetration area may occur
- Osteitis, (septic) loosening, periprosthetic fracture

## Indications

- Prosthesis use less than 50 h per week
- Walking distance less than 2 km
- Frequent skin problems because of the socket
- Uncomfortable sitting
- Loosening of the prosthesis during daily activities
- Complaints of perspiration due to socket/liner

## Contraindications

- Satisfaction with socket prosthesis
- Poorly controlled diabetes or with complications
- Severe bone deformity
- Children and adolescents
- Bone diseases (chronic infection or metastasis)
- Chemotherapy
- Severe vascular diseases
- Pain without a clear cause
- Overweight (body mass index > 30)
- Smoking

## Patient information

It is important that the surgeon informs the patient about:

- Progressive postoperative loading instructions
- Life-long stoma care with water and soap twice daily
- Occurrence of soft tissue irritations in the skin penetration area
- In case of failure, residual limb shortening is expected
- The risk of infection and potential bone infection

## Preoperative work-up

**Fig. 2:**
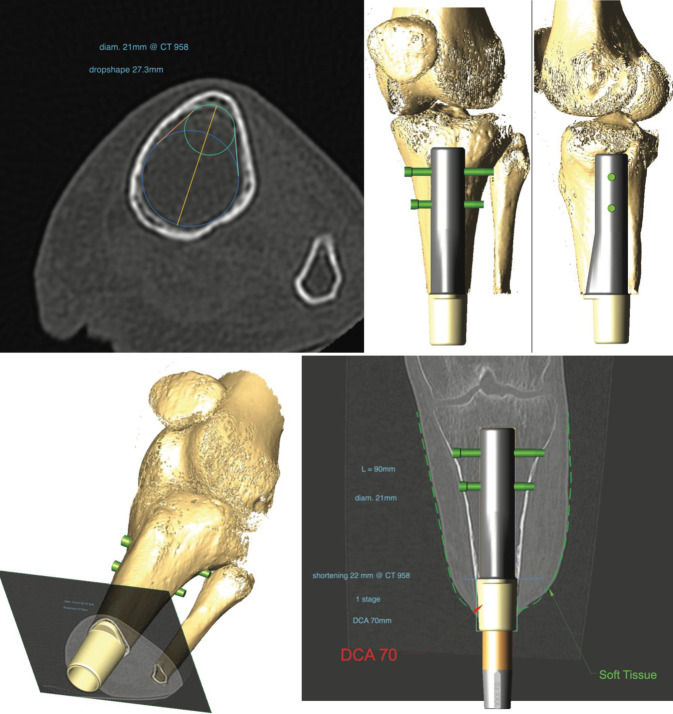
Preoperative surgical planning based on computed tomography (CT) scan images to configure the template tibial remnant

The manufacturer (OTN Implants Arnhem The Netherlands) provides the surgical planning based on computed tomography (CT) scan images to calculate the implant size (Fig. 2).

## Special instruments

- OTNI 13BS and 13TS—reusable surgical instrument basic sets for BADAL X tibia
- OTNI RR01 and RR02 and RR03 rigid reamer sets with diameters 12–23 mm
- DePuy Synthes LCP 5.0 mm screws various sizes according to planning (author’s preference)

## Anesthesia and positioning

**Fig. 3:**
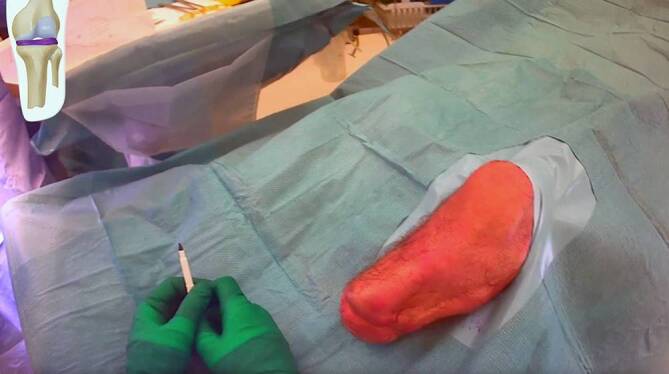
Positioning

The patient is positioned in supine position on a standard radiolucent operating table under general or spinal anesthesia preferably with locoregional nerve block. Perioperative antibiotic prophylaxis is indicated according to institutional standard orthopedic guidelines. A silicon cushion is placed under the knee for stabilization during surgery and optimal viewing with C‑arm. The C‑arm is ideally on the opposite side of the patient. Always test the images obtained with the C‑arm prior to draping. The residual leg is prepared and draped to allow for surgery until just above the knee (Fig. 3).

## Surgical technique

(Figs. 4, 5, 6, 7, 8, 9, 10, 11, 12 and 13)

**Fig. 4:**
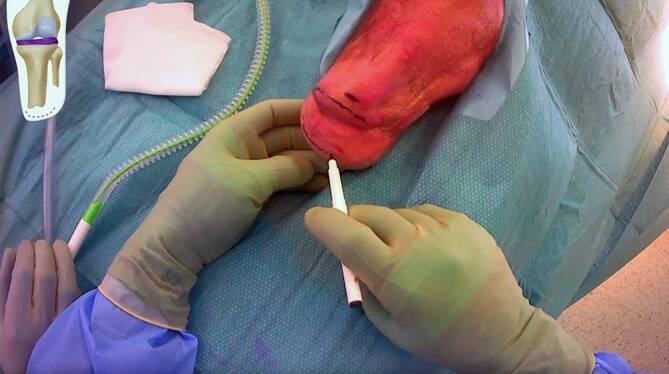
Soft tissue marking: In cases with redundant soft tissues or excessive scars, we tend to mark the resection prior to cutting but it is also an option to perform the soft tissue reduction after implant insertion with the advantage of not having to resect too much

**Fig. 5:**
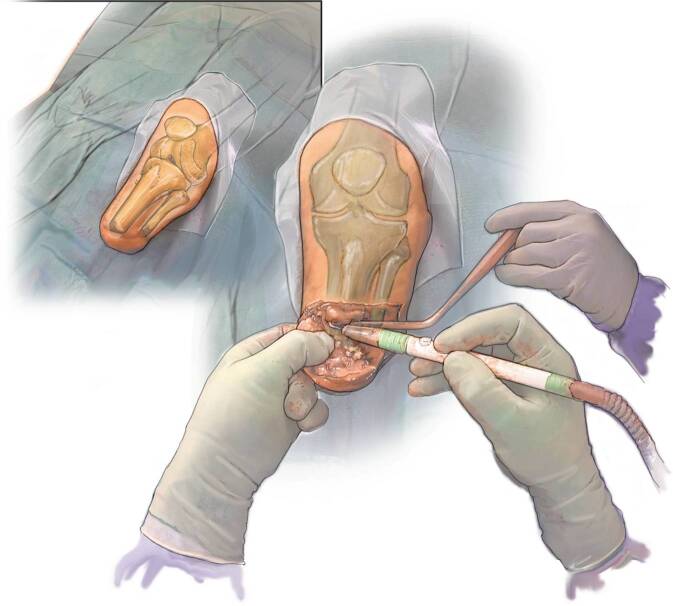
Soft tissue correction and exposure of residual bone: We start with a direct approach to the distal tip of the tibia bone remnant using existing scars as much as possible with removal of redundant skin and soft tissues, exposing the bone and allowing for shortening following the preoperative surgical plan including removal of redundant calf muscles

**Fig. 6:**
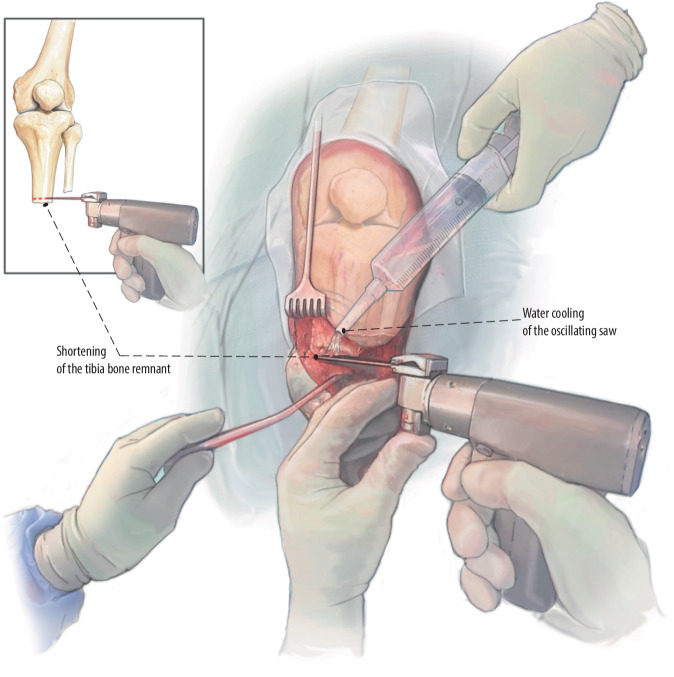
Revision osteotomy with guided shortening: According to the presurgical planning, we perform an osteotomy at the distal end of the tibia to open the medullary canal. In this case, only 12 mm is resected. It is important to water cool the saw to prevent thermal damage to the bone. The fibula is shortened whenever indicated, aiming about 10–20 mm shorter than the level of the tibia

**Fig. 7:**
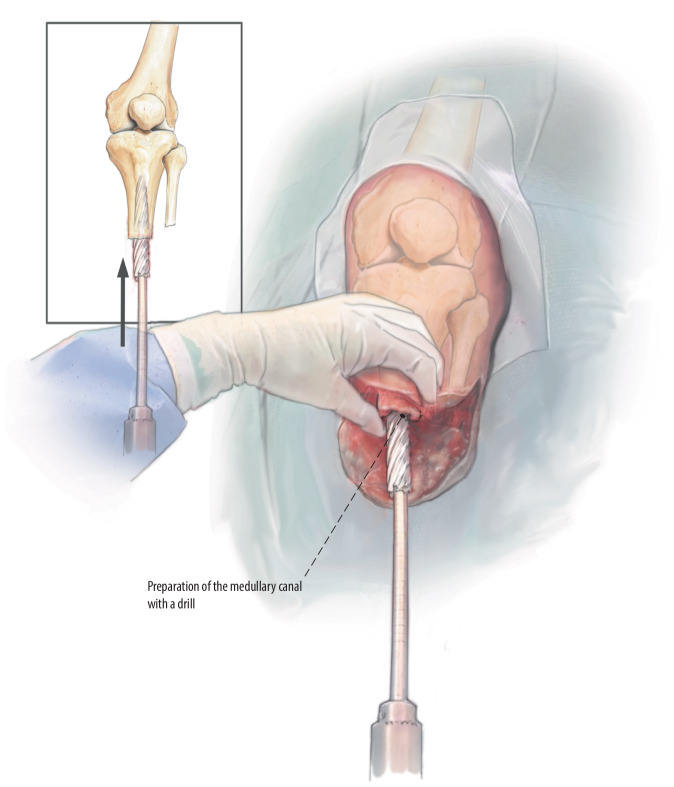
Medullary canal preparation and perpendicular cutoff plane: Preparation of the medullary canal can be performed using the smallest 12 mm rigid drill manually with careful progression; confirm the alignment with fluoroscopy. Further opening of the canal is performed using rigid drills stepwise until reaching the desired size with a power drill. Depending on the bone quality, you may over- or underdrill by one millimeter. Double check the cortical wall after preparation to assess for eventual cracks, after which the distal cut edge is perpendicularly finished by hand or with tip rasps on power drill

**Fig. 8:**
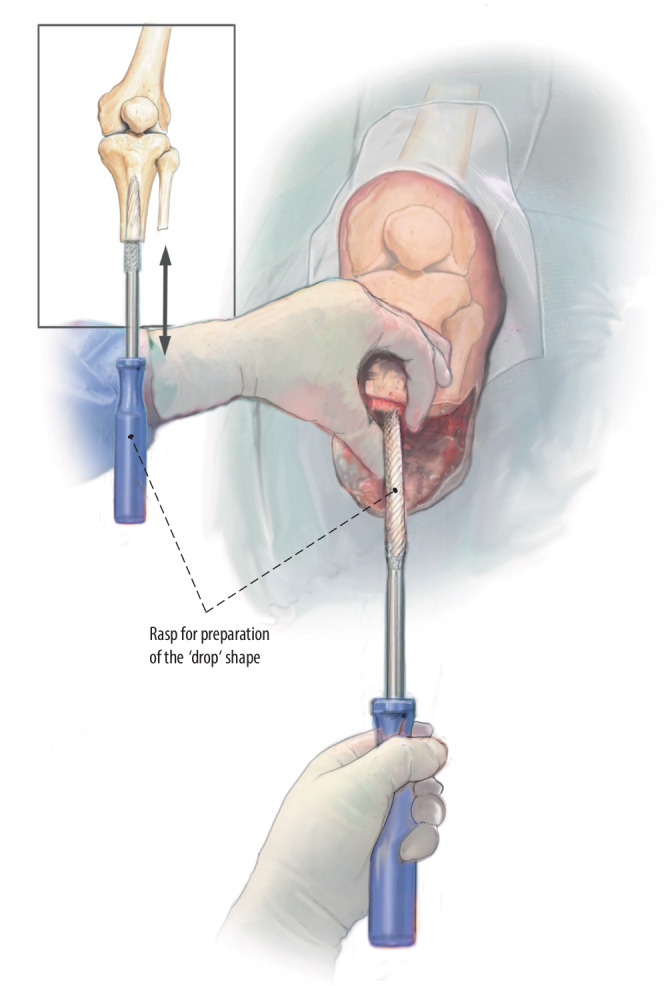
Preparation for drop shape: To prepare the drop shape, we use a manual rasp until the prescribed diameter is achieved, which in this case is 23 mm. Careful assessment must also be made regarding the length of the drop shape and the inclination. To avoid a crack at this level of the tibia crest, do not undersize

**Fig. 9:**
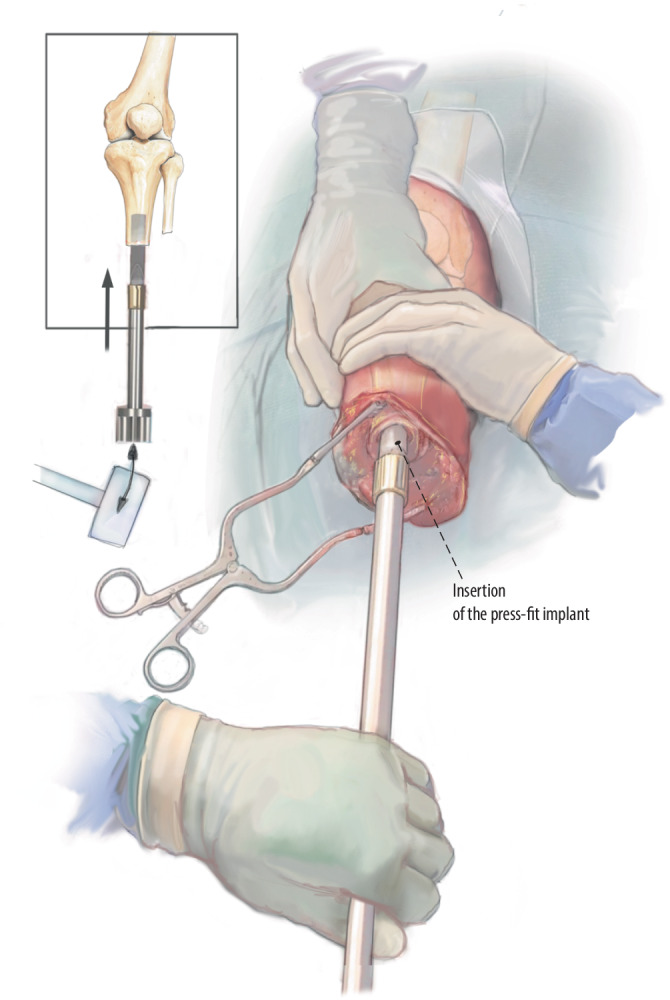
Insertion of intramedullary component: The custom-made stem is mounted on the installer. The implant is then hammered into the bone, and the grip of the press-fit implant is checked, also testing rotational stability. In this case, I drill one extra mm and afterward decide on an adequate fixation. Once this is achieved, we further insert the implant with gentle strikes until the shoulder of the implant is firmly connected to the cut edge of the femoral remnant. When a crack occurs and reamings are available, these reamings are used to fill the crack. Use the C‑arm to guide insertion and finally check the uncomplicated position of the implant with adequate alignment in two directions

**Fig. 10:**
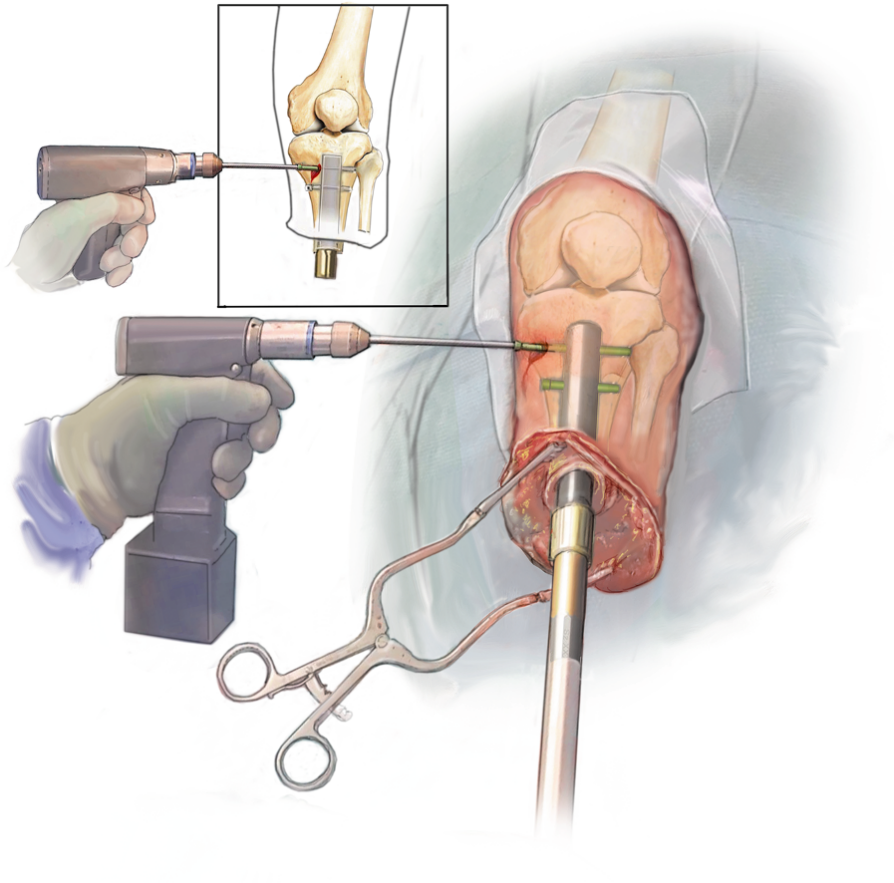
Insertion of transverse locking screws: The C‑arm is positioned to allow free hand placement of the two transverse locking screws in a standard fashion. The large fragment LCP screws are featured with threads on the head of the screw to bury in the tibial cortex. A custom-made disposable guiding device can be ordered if desired

**Fig. 11:**
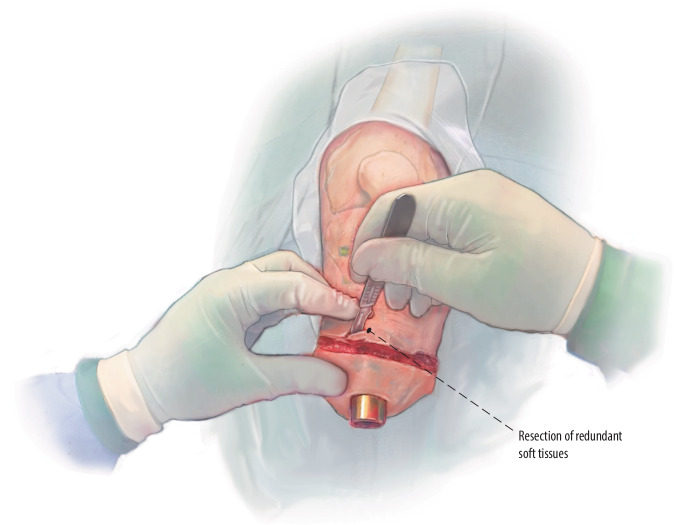
Soft tissue contouring and closure: All soft tissues including skin, subcutaneous fat, and remaining muscles are resected until skin closure is feasible without tension as performed in regular limb amputation surgeries. To create a shallow stoma of 2–3 cm, the subcutaneous fat is removed without thinning of the skin. Remaining muscles are purse-string sutured for hemostasis. The subcutaneous fascia and skin are closed in layers with interrupted sutures covering the head of the implant

**Fig. 12:**
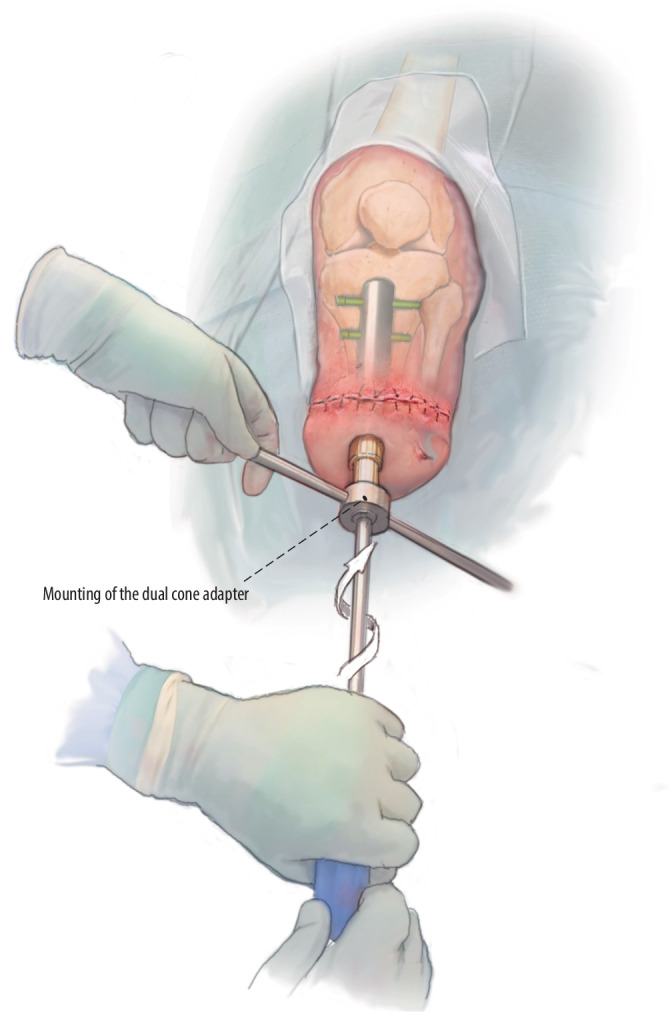
Creation of stoma: A transcutaneous K‑wire is used to identify the correct position of the head of the stem, after which the cannulated corer knife is used to cut a circular defect in the skin and subcutaneous tissue until the head of the implant can be identified and pops out from the stoma. The custom-made dual cone is mounted on the stem after thorough cleaning. The locking screw is inserted and tightened by hand after which the abutment screw is temporarily mounted

**Fig. 13:**
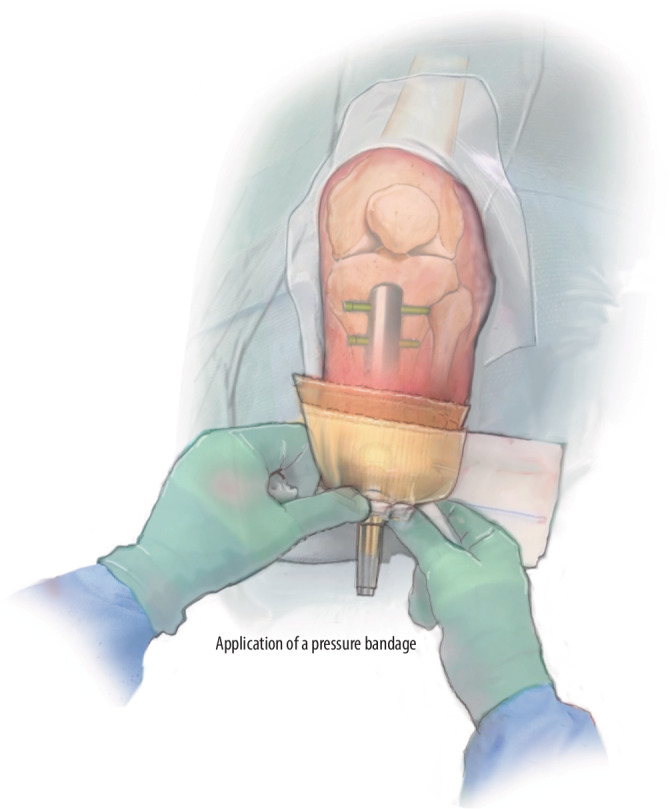
Bandaging: To avoid postoperative bleeding complications, we apply pressure bandage around the residual limb to be removed on postoperative day 2. Extra unfolded gauzes are placed around the dual cone and pressed to control bloody leakage from the stoma during the first postoperative days

## Postoperative management

- Apply pressure bandage for 24 h.
- Inspect wound and stoma at 24 h postoperatively.
- After the 24 h pressure bandage period, cover the stoma with a nonsterile gauze as long as there is some discharge from the stoma.
- Discharge from hospital at day 1 after surgery.
- Limb immobilization and/or anticoagulation therapy is not required (author’s preference).
- Postoperative pain medication according to standard guidelines for knee surgery.
- Give stoma care instructions before discharge from the hospital.
- Instruct the patient to start stoma cleaning with lukewarm tap water and soap twice daily.
- Remove sutures at 10–14 days after surgery.
- Start standard institutional rehabilitation program at 3–4 weeks postoperatively after single-stage surgery. Patients may fully load the prosthesis at the start of the rehabilitation, provided the pain score does not exceed a 5 on a scale from 0–10 [6, 8, 9].

## Errors, hazards and complications

- Intraoperative bone cracks or fractures are treated conservatively or by cerclage depending on implant stability.
- Stoma related complications including; tissue irritation, soft tissue infections are treated in the same way as lower limb bone-anchored prosthesis [3].
- Septic or aseptic implant loosening is diagnosed by X ray and treated with implant removal and antibiotics with staged revision implant whenever indicated.
- Periprosthetic fracture is treated with standard orthopedic surgical or nonoperative treatments similar to lower limb bone anchored prosthesis [7]
- Mechanical failure of the implant is diagnosed by plain X ray and treated with staged implant replacement similar to lower limb bone-anchored prosthesis [11]

## Tips and tricks [5]

- Preoperative implant planning: the procedure should be guided by comprehensive surgical instructions and use a custom-made implant design, with the aim of performing the procedure in a single stage.
- Patient positioning and setup: knee cushion support may be beneficial.
- Soft tissue marking: plan the resection area liberally, and plan the stoma anterior to the surgical approach (if not possible, directly in the wound).
- Soft tissue correction and exposure of residual bone: liberally resect soft tissue redundancy.
- Revision osteotomy with guided shortening: utilize water-cooled power sawing.
- Medullary canal preparation and the perpendicular osteotomy plane: use fluoroscopy to guide drilling.
- A custom dummy stem is available from the manufacturer on request.
- Insertion of the intramedullary component: in case of little resistance, use bone morphogenetic protein‑2 (InductOs; Medtronic) and bone impaction grafting for augmentation.
- Use transverse locking screws for primary stabilization of the implant.
- A custom made disposable guiding device is available for insertion of the transverse locking screws from the manufacturer on request.
- Soft tissue contouring and closure: do not close the muscle fascia over the implant.
- Aperture creation and dual-cone insertion: perform a 2-stage procedure only in cases with bone reconstruction, with the second stage performed after a 10 to 12-week interval.
- Bandage: leave the bandage applied for 24 h.
- Postoperative imaging and follow-up: our institutional follow-up schedule is 6 months, then 1, 2, 5, and 10 years postoperatively.

## Results

We describe the fixed 5‑year follow-up results of 21 individuals with a transtibial amputation treated with tibia TOPS implant. We treated 15 men (71%), with an average age of 37 years (± 16) at amputation and 48 years (± 13) at implantation of TOPS. Amputation etiologies were the following: trauma 14 (67%), dysvascular 3 (14%), infection 2 (10%), or other 2 (10%). Individuals were treated with single-stage surgery 7 times (32%), two-stage surgery 14 times (64%), and with primary amputation combined with TOPS implantation once (5%).

Implant survival of 95.5% was achieved at the 5‑year follow-up. One individual experienced progressive septic implant loosening resulting in a through-knee amputation. The individual had undergone primary transtibial amputation due to dysvascular problems, and preoperative duplex ultrasonography had shown no signs of aorto-iliac occlusive disease. However, repeat exam displayed dysvascular disease progression, with the patient admitting not to have stopped smoking. No bone infection, periprosthetic fracture, intramedullary stem breakage, or aseptic loosening occurred. Nine individuals experienced 12 events of low-grade soft tissue infection, which were all successfully treated with oral antibiotics. Nine individuals also experienced 12 events of high-grade soft tissue infections, successfully treated with oral antibiotics 8 times, and requiring parenteral antibiotics or surgical treatment in 1 and 3 cases, respectively. This resulted in an infection/implant–year used ratio of 0.24. Hypergranulation tissue and stoma tissue redundancy occurred 2 and 4 times, respectively.

Functional outcomes were evaluated using the Questionnaire for Transfemoral Amputees (Q-TFA) Global Score (GS) and Prosthetic Use Score (PUS), showing a significant improvement in outcomes compared to preoperative baseline values using a socket vs postoperative following TOPS treatment: baseline (52.7), 6 months (89.9), 1 year (88.0), 2 years (91.3), and 5 years (88.9). This significant improvement was also observed for the Q‑TFA GS: baseline (38.3), 6 months (71.4), 1 year (79.4), 2 years (76.8), and 5 years (77.6).

## Data Availability

The data that support the findings of this study are available from the corresponding author upon reasonable request.

## Declarations

For this article no studies with human participants or animals were performed by any of the authors. All studies mentioned were in accordance with the ethical standards indicated in each case.
